# The predictive value of multiple artificial intelligence models in axillary lymph node metastasis of breast cancer detected by ultrasound — a network meta-analysis

**DOI:** 10.3389/fonc.2026.1725537

**Published:** 2026-06-29

**Authors:** Zhen Wang, Jian Dong, Yuhang Cheng, Yang Y, Wen Liu, Jun Li, Ting Ma

**Affiliations:** Department of Ultrasound Medicine, the First Affiliated Hospital of Shihezi University, Shihezi, Xinjiang, China

**Keywords:** axillary lymph nodes, cancer metastasis, multiple artificial intelligence, predictive value, ultrasound

## Abstract

**Objective:**

To compare the diagnostic performance of different AI models in predicting axillary lymph node metastasis in breast cancer detected by ultrasound.

**Method:**

A systematic search strategy was conducted in PubMed, Embase, Web of Science, Cochrane Library, CNKI, and Wanfang Data from July 2015 to July 2025 for diagnostic studies on the prediction of axillary lymph node metastasis in breast cancer. Relevant diagnostic accuracy data were extracted and synthesized. A network meta-analysis was performed to compare the performance of different AI models.

**Results:**

This study finally included 9 studies involving 2,813 patients through strict inclusion and exclusion criteria. The predictive value of 11 artificial intelligence models for axillary lymph node metastasis was assessed. The methodological quality of the included studies was good, and there was no obvious publication bias. The pooled diagnostic odds ratio of all diagnostic methods was 10.92, showing statistically significant heterogeneity (I² = 92%, *P* < 0.01). Pooled diagnostic performance showed that Boosting-based algorithm models achieved the highest sensitivity (83.0%) and negative predictive value (86.4%), while the Temporal Interlace Network model achieved the highest specificity (90.0%) and positive predictive value (80.0%). Pairwise comparisons revealed that the sensitivity of Boosting-based algorithm models was significantly higher than that of Support Vector Machines (0.17, 95% CI: 0.01–0.33), and the negative predictive value was significantly higher than that of Support Vector Machines (0.09, 95% CI: 0.01–0.17). No statistically significant differences were observed in the predictive performance of the remaining models.

**Conclusion:**

Boosting-based algorithm models showed a possible signal of favorable and balanced predictive value in ultrasound-based axillary lymph node metastasis, though substantial heterogeneity and inconsistency limit definitive conclusions.

**Systematic review registration:**

https://www.crd.york.ac.uk/prospero/, identifier CRD420251119253.

## Introduction

Breast cancer (BC) is a malignant tumor caused by a combination of factors such as genetics, hormones, age, and living environment (1). Currently, the development of measures such as high-risk population mass screening, neoadjuvant combination therapy, and advanced prevention has reduced the mortality rate of BC and distant metastasis (2, 3). Nonetheless, the unclear mechanisms of BC occurrence and development, insufficient specificity of early diagnosis, challenges in implementing precision therapy, significant differences in prognosis, and heavy socioeconomic burden continue to pose a serious threat to women’s public health safety (4, 5).

BC primarily metastasizes to other vital organs in the human body through lymphatic channels. The heterogeneity of BC metastasis leads to variations in early imaging presentation and poses challenges for precise targeted treatment planning (6). Sentinel lymph node infiltration is a critical indicator of distant metastasis in malignancies (7–9). Ultrasound (US) remains the most widely used clinical method for detecting the morphological characteristics of BC tumors and predicting the risk of axillary lymph node metastasis (ALNM) (10–13). US can predict the presence of tumor metastasis burden within clinically inaccessible axillary lymph nodes, thereby avoiding unnecessary axillary lymph node dissection and guiding precision surgical planning. It is the preferred method for predicting axillary lymph node status and guiding imaging-directed lymph node intervention therapy (14). However, the accuracy (ACC) of US is heavily dependent on the operator’s experience and subjectivity. Additionally, the morphological characteristics of tumors and lymph nodes vary greatly among individuals and are easily affected by anatomical location and interfering factors. Consequently, US has difficulty in predicting axillary lymph node metastasis and tumor development.

The application of machine learning algorithms in US has been extensively explored due to their standardized feature extraction and automated analysis capabilities (15, 16). Previous studies have confirmed the feasibility of machine learning algorithms combined with imaging methods and clinical performance models in predicting ALNM in BC patients (11). A study of Li et al. (17) indicated that the Residual Network (ResNet) model demonstrated the best performance in predicting ALNM in BC patients (AUC = 0.86). Additionally, other related studies suggest that models such as Extreme Gradient Boosting (XGBoost), Support Vector Machines (SVM), and Logistic Regression (LR) may offer the best overall predictive performance for ALNM (10, 18, 19). Each model is based on distinct operational principles and theoretical frameworks (20). Inconsistencies in model performance have hindered the establishment of standardized protocols for selecting optimal models in clinical practice, which may cause some clinical centers to over-rely on a single model, resulting in missed diagnoses or overtreatment, and hindering the standardized implementation of precision treatment strategies. Although the operating principles and theoretical frameworks of different AI models are different, their input features mainly come from ultrasound images, and their outputs are also standardized against the same clinical gold standard of ALNM confirmed by pathology. Therefore, although the feature extraction mechanisms of different models may capture non overlapping biological or imaging features and introduce heterogeneity in indirect comparisons, they are comparable in diagnostic performance indicators.

To explore the comparative diagnostic performance of multiple AI models for ALNM and to identify potential signals of favorable performance, this study conducted a network meta-analysis of the predictive efficacy of multiple artificial intelligence models in ALNM in BC patients. The consistency of existing predictive evidence has been analyzed. By directly or indirectly integrating data, the relative efficacy of multiple models has been quantitatively ranked to generate hypotheses about which models may warrant further investigation. This study provides hypothesis-generating comparative signals that may inform the design of future validation studies.

## Methods

### Search strategy

This study systematically searched for studies on AI prediction of ALNM published from July 2015 to July 2025. The search databases included PubMed, Embase, Web of Science, Cochrane Library, CNKI, Wanfang Data, etc. Two researchers conducted a comprehensive screening of relevant studies using a combination of MeSH terms and free terms. The main search terms included: “Artificial Intelligence”, “Computer Reasoning”, “Axillary lymph nodes”, “Ultrasonography”, and “Diagnostic Ultrasound”. No language restrictions were applied to the search. This study also conducted a manual search of the references of relevant studies.

### Inclusion and exclusion criteria

This systematic review and network meta-analysis was performed in accordance with the PRISMA statement (CRD420251119253). The PICOS framework was defined as follows: P (population): patients with BC; I (index test): AI models based on ultrasound images; C (comparator): other AI models; O (outcome): diagnostic performance; S (study design): diagnostic accuracy studies comparing at least two AI models.

To ensure the rigor of the study, strict inclusion and exclusion criteria were established. The inclusion criteria were as follows: ① Patients with pathologically confirmed BC. ② Clear reference criteria such as surgery or biopsy confirmed whether the patient have ALNM. ③ Two or more artificial intelligence models based on US images were used to predict whether the patients have ALNM. ④ Patients for whom diagnostic-related data from the model predictions could be obtained directly or indirectly. ⑤ All AI models were required to be based on conventional ultrasound images (B-mode and/or Doppler). The exclusion criteria were as follows: ① The content of the studies is inconsistent with the direction of this study: research content unrelated to artificial intelligence prediction of ALNM in BC, patients with confirmed metastasis, patients who underwent biopsy or interventional therapy prior to US examination, etc. ② Missing information: Inability to obtain the full text of the study, lack of clear inclusion/exclusion criteria or pathological reference standards in the full text, inability to obtain accurate prediction data, etc. ③ Study type: Exclude letters to the editor, case discussions, reviews, lecture submissions, etc. Two researchers independently read the studies retrieved in strict accordance with the inclusion and exclusion criteria and selected those eligible for diagnostic accuracy studies. In case of disagreement, a senior review researcher made the final decision after consultation.

### Methodological quality assessment

To systematically assess the impact of included studies on the overall quality and bias of the research, the researchers carefully read the full texts and conducted a methodological quality assessment of the included diagnostic tests using the Quality Assessment of Diagnostic Accuracy Studies-2 (QUADAS-2) scale. To avoid assessment errors caused by personal subjective factors, two researchers independently assessed the risk of bias in the included studies. In the event of any discrepancies, a senior review researcher made the final decision through consultation.

### Data collection

The researchers collected basic data from each study, including: study authors, year of publication, number of cases participating in the study, number of axillary lymph node-positive and node-negative cases, reference standards, predictive models, etc. Importantly, the researchers specifically extracted predictive diagnostic data from each AI model.

### Statistical analysis

Based on the total number of cases, the number of axillary lymph node positive and negative cases, this study derived the true positive, false positive, false negative, true negative, sensitivity (SEN), specificity (SPE), accuracy, positive predictive value (PPV), and negative predictive value (NPV) data for each included study. A frequentist network meta-analysis was performed using Stata MP/18 with the *network* suite. A random-effects model with restricted maximum likelihood estimation was assumed. The network meta-analysis was conducted within a multivariate framework to preserve the within-study correlation between sensitivity and specificity. Heterogeneity was assessed using the I² statistic, if I² > 50%, a random-effects model was used to integrate the data to account for variability between studies. Network geometry was visualized using *network map*. To address the potential heterogeneity caused by differences in model architecture, inconsistency was assessed using global inconsistency tests (*network meta i*). When P > 0.05, a consistency model (network meta c) was fitted; when P < 0.05, we explored potential sources of inconsistency through subgroup and sensitivity analyses.

Given that multiple AI models were often evaluated in the same patient cohort, their diagnostic accuracy estimates were correlated. To account for this, we used a multivariate meta-analysis model with a block-diagonal variance-covariance matrix, approximating covariances using the method of Wei and Higgins (2013). Sensitivity analyses assuming different correlation strengths (ρ = 0.2, 0.6, 0.8) were conducted to assess robustness. If the reported data were insufficient to reconstruct the covariance matrix, this limitation is acknowledged.

A funnel plot was constructed and Deeks’ funnel plot asymmetry test (*netfunnel*) was performed to examine publication bias. The relative predictive performance of the models was determined by ranking them in descending order based on the Surface Under the Cumulative Ranking curve (SUCRA) to assess the magnitude of their predictive effects on ALNM. SUCRA rankings were derived from 5,000 resampling iterations.

Model fit was evaluated by comparing the AIC or residual deviance between consistency and inconsistency models, supplemented by leverage plots and residual deviance. Pairwise comparisons were presented as forest plots and league tables (*intervalplot, netleague*). Forest plots and league tables were constructed to illustrate the comparative predictive efficacy and validity of the models. Conventional diagnostic meta-analysis was performed using *midas* and *metan*.

In addition, we conducted subgroup analysis based on model categories to reduce the risk of misleading comparisons across different algorithm paradigms. To address heterogeneity arising from different algorithmic architectures, models were categorized into three groups: Boosting-based, CNN-based (ResNet, Inception, VGG, TIN), and traditional machine learning (SVM, RF, DT, KNN, LR, NB). Pooled diagnostic performance and within-group heterogeneity (I²) were calculated for each group. Meta-regression was used to compare performance across groups. To analyze the transitivity and comparability of network meta-analysis, we evaluated the impact of study design, reference standards, sample size, and ALNM prevalence in various studies. To address potential violations, we conducted sensitivity analysis by excluding studies with inconsistent reference standards (13). Meta-regression was performed based on ALNM prevalence.

## Results

### Basic studies information

**Figure 1:**
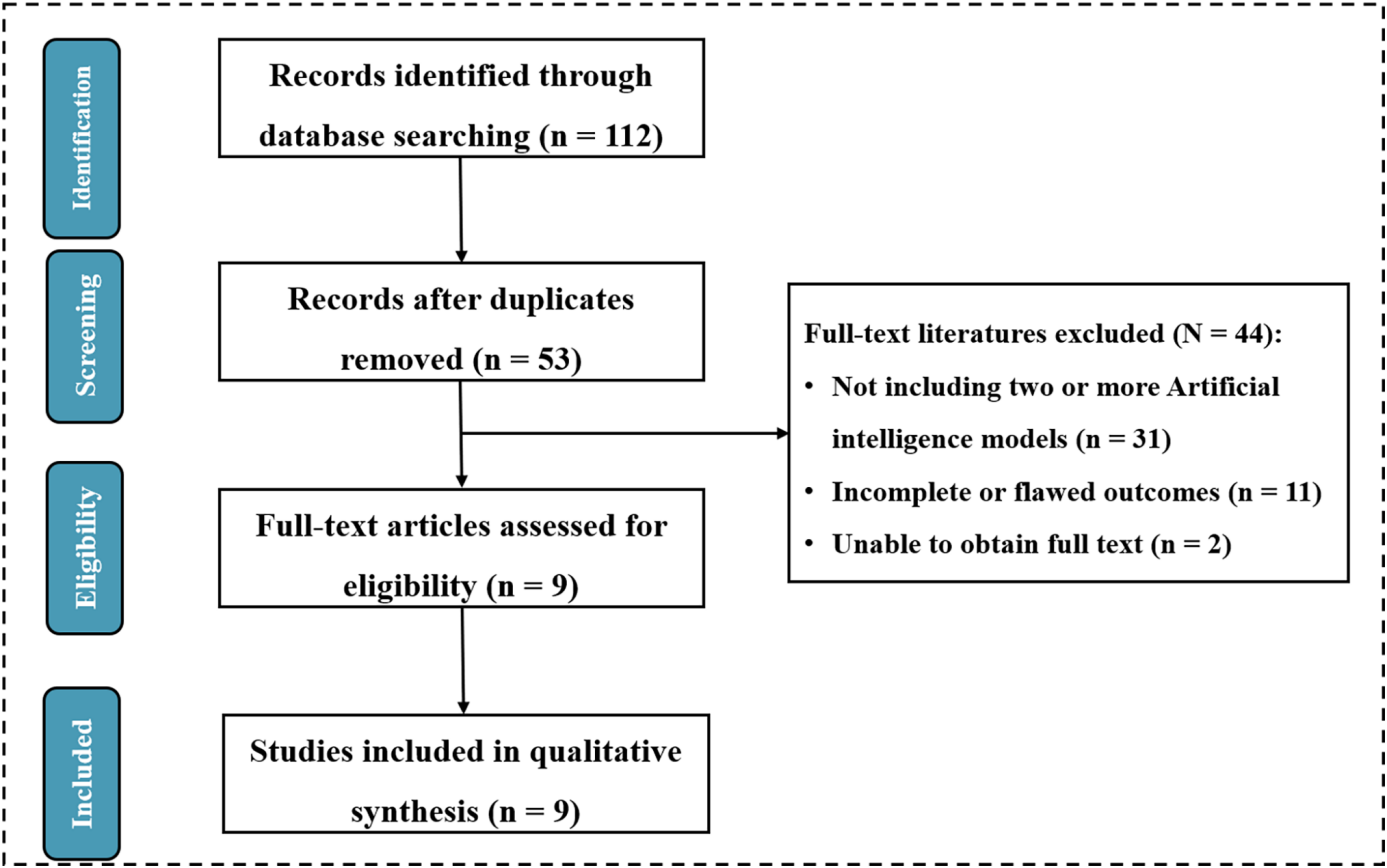
Flow chart of studies retrieval and screening.

A total of 112 potentially relevant studies were retrieved from the database, of which 59 duplicates were excluded, leaving 53 studies. The researchers carefully read the 53 studies according to the inclusion and exclusion criteria, and ultimately included 9 studies involving 2,813 patients (13, 17, 18, 21–26). The full reproducible search strategies for each database, including search terms, Boolean operators, field tags, time restrictions, and execution date (July 25, 2025) are provided in **Supplementary Material 1**. All included patients had received no prior treatment before ultrasound examination. Of the nine included studies, six explicitly enrolled only female patients. Just one study reported three male patients (18) and the remaing two studies did not report sex distribution (17, 23). The specific search process is shown in **Figure 1**. Among the 9 included studies, 3 were prospective studies and 6 were retrospective studies. A total of 11 AI models were included in the study, including Boosting-based algorithm models, Decision Tree (DT), Inception, K-Nearest Neighbor (KNN), LR, Naive Bayesian (NB), Random Forest (RF), ResNet, SVM, TIN and Visual Geometry Group (VGG). The baseline information of the included studies is summarized in **Table 1**.

**Table 1:** Baseline characteristics of the included studies.

Author	Year	Country	Study type	Number of patients	Positive axillary lymph nodes	Negative axillary lymph nodes	Gold standard	Models
Gaosen Zhang	2022	China	retrospective study	952*	394	558	surgery pathology(305) percutaneous needle biopsy(597)	SVM
								Boosting
								RF
								LR
								NB
								KNN
								ResNet
Wei-Bin Li	2023	China	prospective study	61	34	27	surgical pathology	TIN
								Inception
								VGG
								ResNet
Pengfei Sun	2024	China	prospective study	240	88	152	percutaneous needle biopsy	Boosting
								KNN
								NB
								RF
								LR
								DT
								SVM
Si-Rui Wang	2024	China	retrospective study	224	88	136	surgical pathology	LR
								DT
								Boosting
								KNN
								SVM
								RF
Li-Qiang Zhou	2019	China	retrospective study	178	90	88	surgical pathology	Inception
								ResNet
Weimin Xu	2025	China	retrospective study	193	71	122	surgical pathology	RF
								Boosting
Xueyi Zheng	2020	China	prospective study	584	147	337	surgical pathology	ResNet
								Inception
								VGG
Meiying Yan	2024	China	retrospective study	282	87	195	surgical pathology	LR
								NB
								SVM
								KNN
								Boosting
Ranze Cai	2024	China	retrospective study	99	33	66	surgical pathology	RF
								SVM
								Boosting

*Zhang et al. (2022): 902 primary cohort (305 surgery + 597 biopsy) + 50 validation cohort (pathology), total 952 patients. *SVM, Support Vector Machine; RF, Random Forest; DT, Decision Tree; NB, Naive Bayes; LR, Logistic Regression; KNN, K-Nearest Neighbors; ResNet, Residual Network; Inception, Inception Network; VGG, Visual Geometry Group Network; TIN, Temporal Interlace Network and Boosting, Boosting-based algorithm models.

### Quality assessment

**Figure 2:**
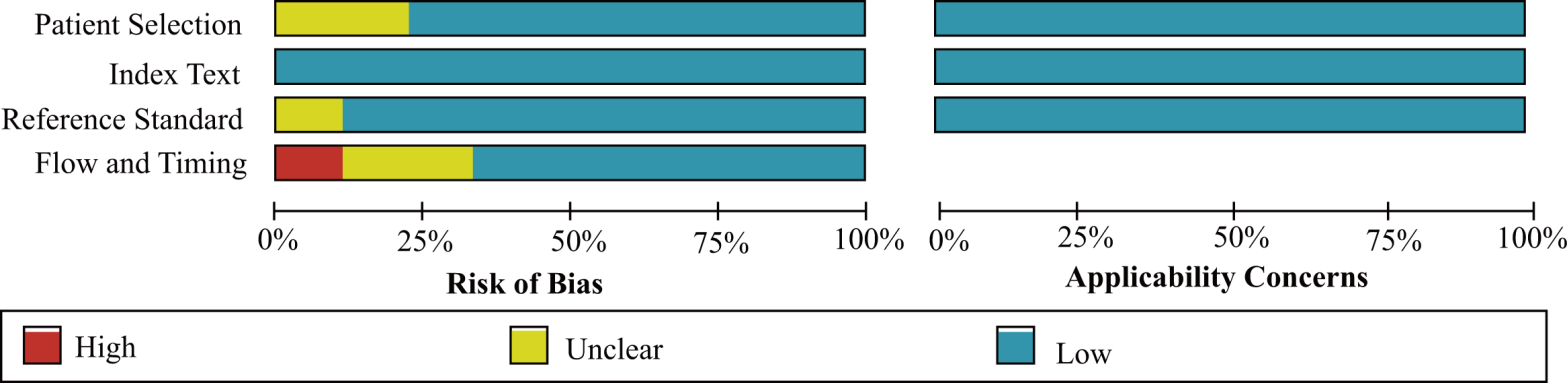
QUADAS 2 criteria of the included studies.

This study assessed the quality of nine studies using the QUADAS-2 scale. The included studies demonstrated high quality, with specific risk proportions shown in **Figure 2**. Except for the studies of Gaosen Zhang et al. (13), which used inconsistent reference standards (305 patients underwent surgical pathology and 558 patients underwent percutaneous needle biopsy), no other studies showed obvious high-risk factors.

### Bias risk and consistency test

**Figure 3:**
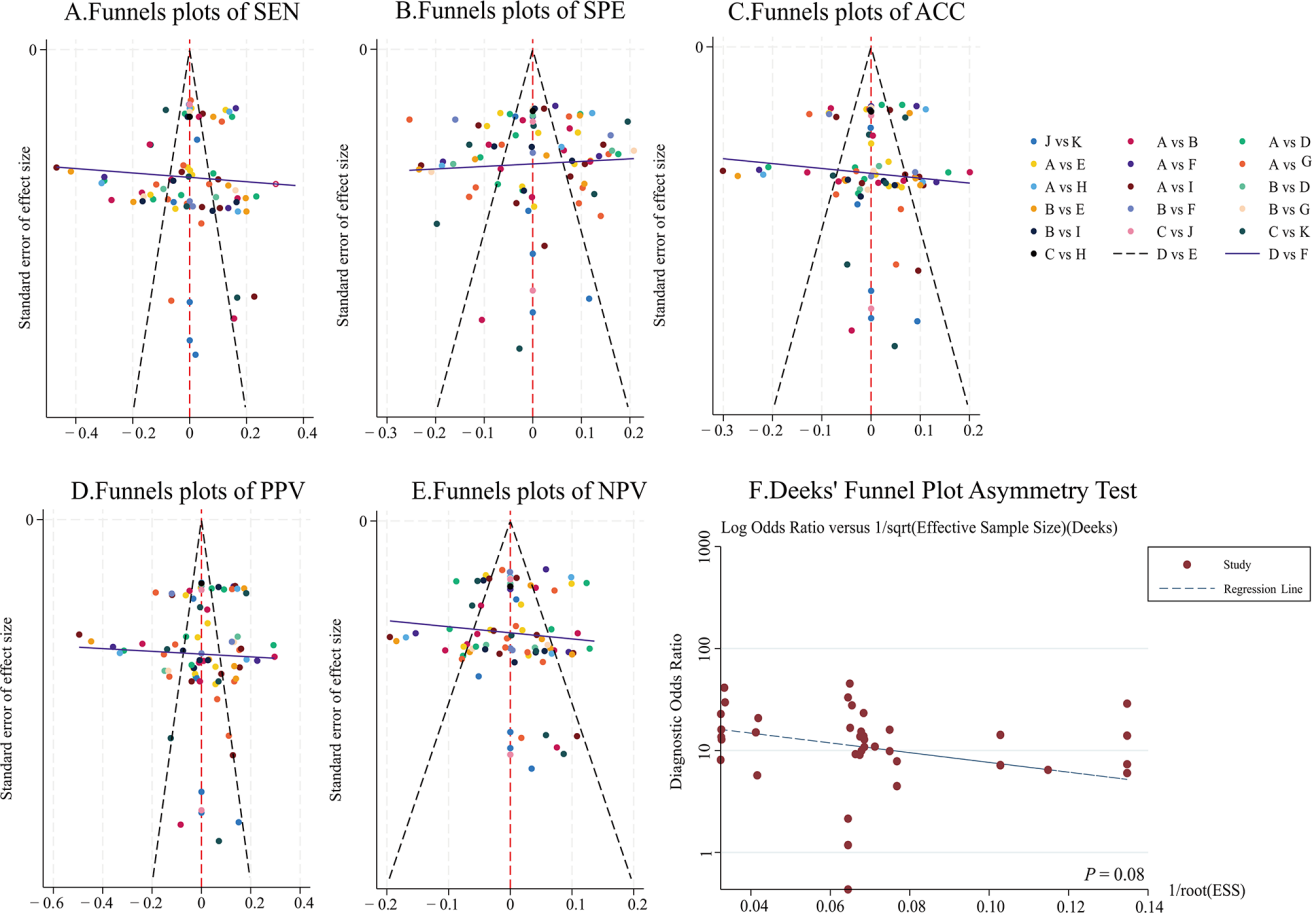
Risk of bias analysis. **(A)** Funnels plots of SEN. **(B)** Funnels plots of SPE. **(C)** Funnels plots of ACC. **(D)** Funnels plots of PPV. **(E)** Funnels plots of NPV. **(F)** Deeks' Funnel plot asymmetry test.

A funnel plot of the diagnostic data from the nine studies showed good symmetry. The Deeks’ Funnel Plot Asymmetry Test yielded *P* = 0.08 > 0.05, indicating no significant publication bias, as shown in **Figure 3**. Consistency tests for all diagnostic indicators using Stata MP/18 yielded *P* < 0.05. Model fit assessment using AIC revealed that the inconsistency model yielded a substantially lower value than the consistency model (AIC: 142.3 vs. 156.8), indicating statistically significant inconsistency between direct and indirect comparisons.

### Network node diagram

**Figure 4:**
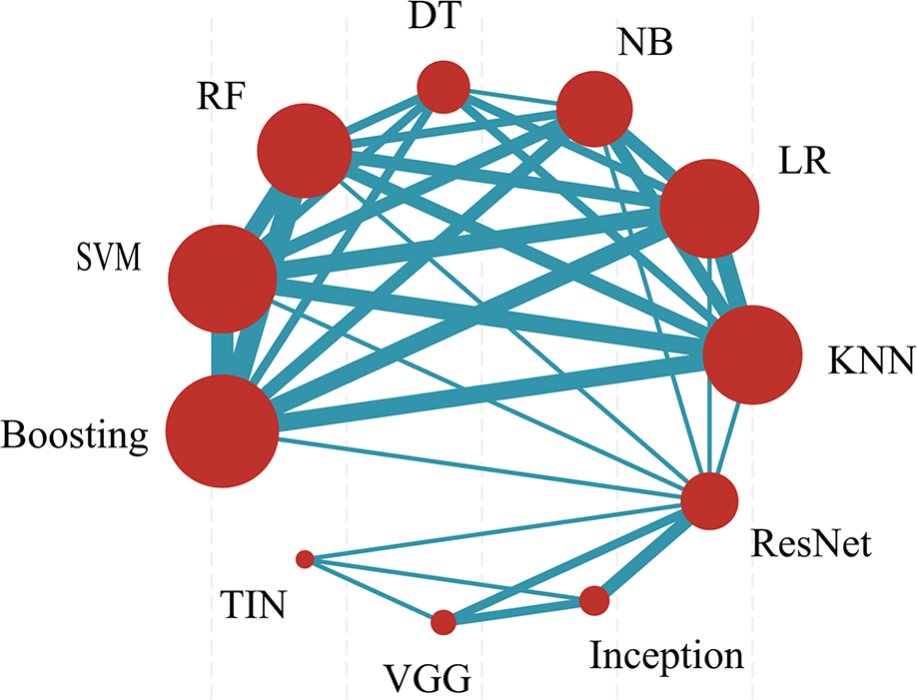
Network diagrams.

This study summarized the connections between all included artificial intelligence models, as shown in **Figure 4**. The study found that Boosting-based algorithm models and SVM model had the largest nodes and included the largest sample sizes. TIN models had the smallest nodes and included the smallest sample sizes. The connections between Boosting-based algorithm models and SVM model, as well as between Boosting-based algorithm models and RF models were the thickest, indicating the closest direct connections between models. The overall interactivity between all models, both direct and indirect, was close.

### Diagnostic performance of the models

**Figure 5:**
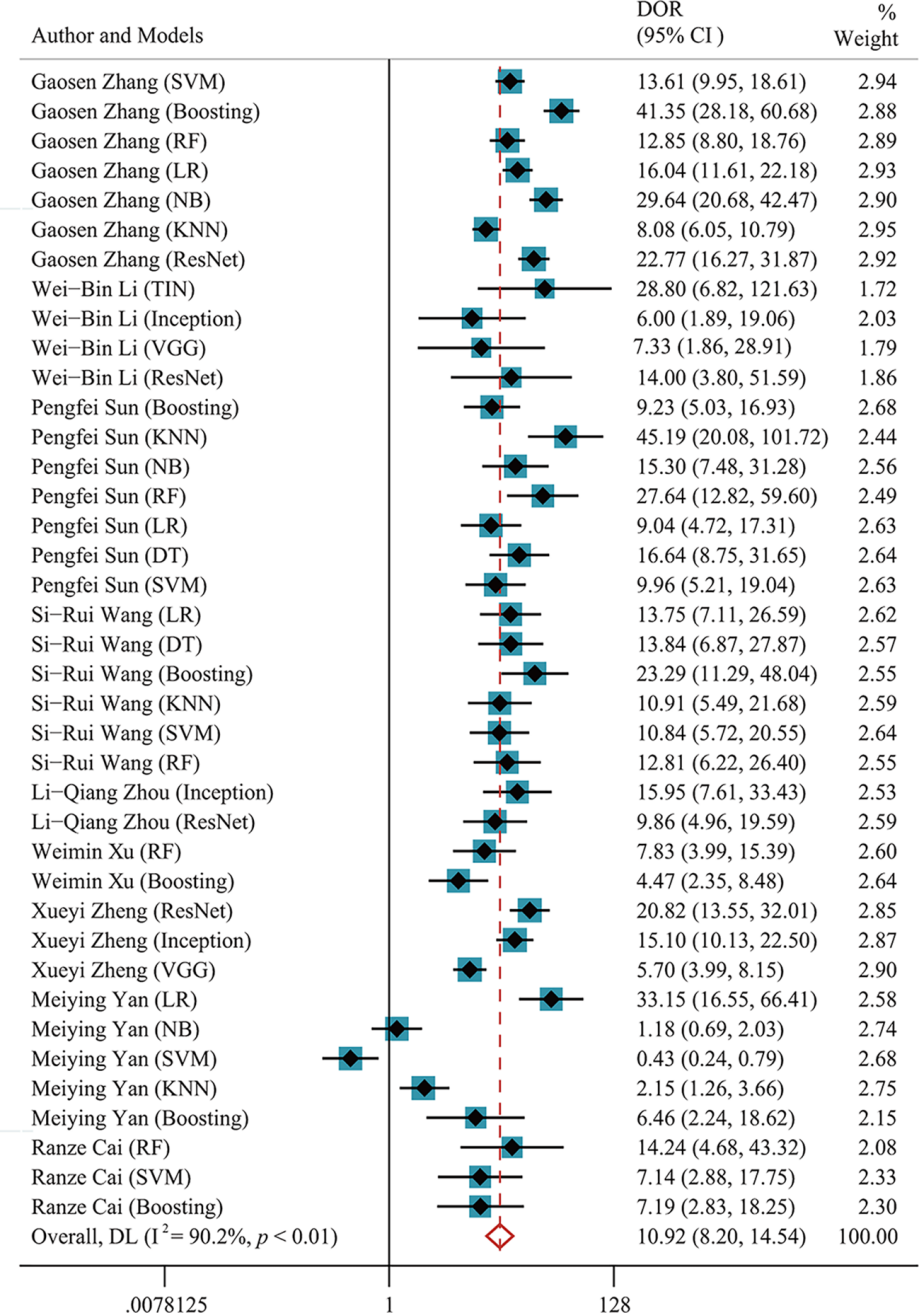
Diagnostic ratio.

The overall diagnostic odds ratio (DOR) across all studies was 10.92, which was statistically significant (I² = 92%, *P* < 0.01) (see **Figure 5**). Except for the NB model (DOR: 1.18, 95% CI: 0.69-2.03) and SVM model (DOR: 0.43, 95% CI: 0.24-0.79) in the studies of Meiying Yan et al. (26), the DOR of all other models was greater than 1.

Pooled diagnostic performance of individual AI models and AI models groups.

The pooled diagnostic performance for each model is summarized in **Table 2**. Boosting achieved the highest pooled SEN (83.0%, 95% CI: 73.4-89.6%) and NPV (86.4%, 95% CI: 81.0-90.6%). TIN achieved the highest pooled SPE (90.0%, 95% CI: 75.7-97.1%), ACC (85.3%, 95% CI: 73.8-93.0%), and PPV (80.0%, 95% CI: 55.7-94.3%). SVM showed the lowest pooled SEN (65.6%, 95% CI: 57.0-73.4%) and NPV (77.2%, 95% CI: 71.7-81.9%). RF showed the lowest pooled SPE (67.1%, 95% CI: 54.4-77.7%).

**Table 2:** Pooled diagnostic performance of individual AI models.

Model	Studies (n)	SEN (%) (95% CI)	SPE (%) (95% CI)	ACC (%) (95% CI)	PPV (%) (95% CI)	NPV (%) (95% CI)
Boosting	6	83.0 (73.4-89.6)	84.7 (78.9-89.2)	84.0 (79.5-87.8)	81.2 (74.0-86.8)	86.4 (81.0-90.6)
RF	5	83.5 (74.6-89.7)	67.1 (54.4-77.7)	72.6 (66.2-78.3)	64.4 (57.2-71.1)	85.2 (77.0-90.9)
LR	4	79.1 (71.7-85.0)	80.6 (73.1-86.5)	80.0 (76.1-83.5)	74.2 (68.2-79.4)	84.6 (80.1-88.3)
SVM	5	65.6 (57.0-73.4)	79.0 (72.6-84.3)	73.3 (68.1-78.0)	68.3 (60.0-75.7)	77.2 (71.7-81.9)
KNN	4	71.5 (63.7-78.3)	76.3 (67.2-83.5)	74.1 (67.1-80.1)	70.5 (60.1-79.1)	77.3 (71.3-82.4)
NB	3	70.4 (52.8-83.5)	83.9 (76.5-89.3)	79.1 (68.5-86.9)	70.7 (52.7-84.0)	83.8 (76.7-89.1)
ResNet	5	79.7 (74.9-83.8)	78.0 (72.9-82.4)	78.7 (75.8-81.4)	75.2 (71.0-79.0)	82.2 (78.6-85.3)
DT	3	70.6 (55.9-82.0)	84.2 (75.0-90.5)	78.0 (69.9-84.4)	78.8 (67.0-87.1)	77.4 (70.3-83.3)
Inception	4	76.4 (71.0-81.1)	80.5 (71.3-87.3)	78.8 (74.5-82.6)	71.8 (64.5-78.2)	83.8 (79.2-87.6)
VGG	3	71.1 (58.5-81.2)	74.0 (58.5-85.1)	72.8 (64.2-80.0)	64.0 (50.9-75.4)	80.0 (71.5-86.4)
TIN*	1	76.2 (52.8-91.8)	90.0 (75.7-97.1)	85.3 (73.8-93.0)	80.0 (55.7-94.3)	87.8 (73.8-95.9)

*TIN was reported in only one study; its 95% CI was calculated using the exact Clopper-Pearson method. All other pooled estimates were derived from random-effects model.

The 11 models were categorized into three groups: Boosting-based, CNN-based, and traditional ML. Boosting-based algorithms achieved the highest pooled SEN (79.6%) and NPV (87.1%), while CNN-based models demonstrated the highest pooled SPE (84.5%) and PPV (79.3%). Traditional ML models showed the lowest pooled SEN (68.2%) and SPE (78.9%). Regarding within-group heterogeneity, traditional ML models exhibited the highest heterogeneity across all diagnostic metrics (I² = 91%), whereas Boosting-based algorithms showed relatively lower heterogeneity (I² = 63%) (see **Table 3**).

**Table 3:** Subgroup analysis results about AI models groups and heterogeneity testing.

Model groups	Sample size (n)	SEN		SPE		ACC		PPV		NPV	
		Pooled value (%)	I² (%)	Pooled value (%)	I² (%)	Pooled value (%)	I² (%)	Pooled value (%)	I² (%)	Pooled value (%)	I² (%)
Boosting-based	1,990	79.6	68	83.2	72	81.5	65	73.5	70	87.1	63
CNN-based	1668	73.4	82	84.5	79	78.6	80	79.3	75	79.8	77
Traditional ML	4431	68.2	91	78.9	88	73.8	89	64.7	86	81.2	84

The sample size is the model sample interaction count (the same patient can contribute multiple models). I ²>75% indicates high heterogeneity.

SUCRA ranking of axillary lymph node prediction models.

This study used SUCRA to rank the diagnostic performance of different artificial intelligence models in predicting ALNM (see **Table 4**). Boosting-based models ranked highest for SEN (SUCRA = 71.9) and NPV (SUCRA = 80.9), while TIN ranked highest for SPE (SUCRA = 79.6) and PPV (SUCRA = 82.2).

**Table 4:** SUCRA ranking of axillary lymph node prediction models.

Models	SUCRA values				
	SEN	SPE	ACC	PPV	NPV
Boosting	71.9	58.1	69.9	61.2	80.9
DT	38.4	73.3	42.7	62.0	35.4
Inception	54.8	53.1	54.3	56.6	49.8
KNN	44.7	36.3	55.7	39.9	36.3
LR	62.3	55.4	66.6	60.1	68.3
NB	23.3	52.8	36.6	29.5	48.0
RF	66.2	23.0	46.5	46.1	72.2
ResNet	48.1	63.6	56.4	66.3	39.6
SVM	21.4	37.5	24.7	18.8	27.4
TIN	56.5	79.6	77.4	82.2	50.8
VGG	59.5	17.3	19.2	27.3	35.4

Red: highest value; Blue: Lowest value.SVM, Support Vector Machine; RF, Random Forest; DT, Decision Tree; NB, Naive Bayes; LR, Logistic Regression; KNN, K-Nearest Neighbors; ResNet, Residual Network; Inception, Inception Network; VGG, Visual Geometry Group Network; TIN, Temporal Interlace Network; Boosting, Boosting-based algorithm models.

Pairwise comparisons between models:

Pairwise comparisons of all artificial intelligence models revealed that the SEN of Boosting-based algorithm models was significantly higher than that of SVM (0.17, 95% CI: 0.01–0.33). The NPV of Boosting-based algorithm models was significantly higher than that of SVM (0.09, 95% CI: 0.01–0.17). No significant differences were observed in the predictive performance of the remaining models (see **Tables 5**, **6**).

**Table 5:** League table on SEN.

Boosting	RF	LR	TIN	VGG	Inception	ResNet	KNN	DT	NB	SVM
**Boosting**	-0.02 (-0.18,0.14)	-0.03 (-0.21,0.14)	-0.02 (-0.47,0.44)	-0.02 (-0.41,0.36)	-0.05 (-0.41,0.32)	-0.07 (-0.37,0.22)	-0.09 (-0.26,0.09)	-0.11 (-0.34,0.11)	-0.17 (-0.36,0.03)	-0.17 (-0.33,-0.01)
0.02 (-0.14,0.18)	**RF**	-0.01 (-0.20,0.17)	-0.00 (-0.46,0.46)	-0.01 (-0.40,0.39)	-0.03 (-0.40,0.34)	-0.05 (-0.35,0.25)	-0.07 (-0.25,0.12)	-0.10 (-0.33,0.13)	-0.15 (-0.35,0.06)	-0.15 (-0.32,0.02)
0.03 (-0.14,0.21)	0.01 (-0.17,0.20)	**LR**	0.01 (-0.45,0.47)	0.01 (-0.39,0.40)	-0.01 (-0.39,0.36)	-0.04 (-0.34,0.26)	-0.05 (-0.24,0.13)	-0.08 (-0.31,0.15)	-0.13 (-0.33,0.07)	-0.14 (-0.31,0.04)
0.02 (-0.44,0.47)	0.00 (-0.46,0.46)	-0.01 (-0.47,0.45)	**TIN**	-0.01 (-0.36,0.35)	-0.03 (-0.37,0.32)	-0.05 (-0.40,0.29)	-0.07 (-0.53,0.39)	-0.10 (-0.58,0.39)	-0.15 (-0.61,0.32)	-0.15 (-0.61,0.31)
0.02 (-0.36,0.41)	0.01 (-0.39,0.40)	-0.01 (-0.40,0.39)	0.01 (-0.35,0.36)	**VGG**	-0.02 (-0.28,0.23)	-0.05 (-0.30,0.21)	-0.06 (-0.46,0.33)	-0.09 (-0.52,0.34)	-0.14 (-0.54,0.26)	-0.14 (-0.54,0.25)
0.05 (-0.32,0.41)	0.03 (-0.34,0.40)	0.01 (-0.36,0.39)	0.03 (-0.32,0.37)	0.02 (-0.23,0.28)	**Inception**	-0.02 (-0.24,0.19)	-0.04 (-0.41,0.33)	-0.07 (-0.47,0.34)	-0.12 (-0.50,0.26)	-0.12 (-0.49,0.25)
0.07 (-0.22,0.37)	0.05 (-0.25,0.35)	0.04 (-0.26,0.34)	0.05 (-0.29,0.40)	0.05 (-0.21,0.30)	0.02 (-0.19,0.24)	**ResNet**	-0.02 (-0.32,0.29)	-0.04 (-0.38,0.30)	-0.09 (-0.40,0.21)	-0.10 (-0.40,0.20)
0.09 (-0.09,0.26)	0.07 (-0.12,0.25)	0.05 (-0.13,0.24)	0.07 (-0.39,0.53)	0.06 (-0.33,0.46)	0.04 (-0.33,0.41)	0.02 (-0.29,0.32)	**KNN**	-0.03 (-0.26,0.21)	-0.08 (-0.28,0.12)	-0.08 (-0.26,0.10)
0.11 (-0.11,0.34)	0.10 (-0.13,0.33)	0.08 (-0.15,0.31)	0.10 (-0.39,0.58)	0.09 (-0.34,0.52)	0.07 (-0.34,0.47)	0.04 (-0.30,0.38)	0.03 (-0.21,0.26)	**DT**	-0.05 (-0.30,0.20)	-0.05 (-0.28,0.17)
0.17 (-0.03,0.36)	0.15 (-0.06,0.35)	0.13 (-0.07,0.33)	0.15 (-0.32,0.61)	0.14 (-0.26,0.54)	0.12 (-0.26,0.50)	0.09 (-0.21,0.40)	0.08 (-0.12,0.28)	0.05 (-0.20,0.30)	**NB**	-0.00 (-0.20,0.19)
**0.17 (0.01,0.33)**	0.15 (-0.02,0.32)	0.14 (-0.04,0.31)	0.15 (-0.31,0.61)	0.14 (-0.25,0.54)	0.12 (-0.25,0.49)	0.10 (-0.20,0.40)	0.08 (-0.10,0.26)	0.05 (-0.17,0.28)	0.00 (-0.19,0.20)	**SVM**

Data are differences in SEN (column-defining model minus row-defining model) with 95% confidence intervals. A positive value indicates that the column model has higher diagnostic performance than the row model. Bold font indicates statistical significance. SVM, Support Vector Machine; RF, Random Forest; DT, Decision Tree; NB, Naive Bayes; LR, Logistic Regression; KNN, K-Nearest Neighbors; ResNet, Residual Network; Inception, Inception Network; VGG, Visual Geometry Group Network; TIN, Temporal Interlace Network; Boosting, Boosting-based algorithm models.

**Table 6:** League table on NPV.

Boosting	RF	LR	TIN	Inception	NB	ResNet	KNN	VGG	DT	SVM
**Boosting**	-0.02 (-0.09,0.06)	-0.02 (-0.11,0.06)	-0.04 (-0.26,0.19)	-0.06 (-0.23,0.12)	-0.05 (-0.15,0.04)	-0.07 (-0.21,0.06)	-0.07 (-0.16,0.01)	-0.09 (-0.27,0.10)	-0.08 (-0.19,0.03)	-0.09 (-0.17,-0.01)
0.02 (-0.06,0.09)	**RF**	-0.01 (-0.10,0.08)	-0.02 (-0.25,0.20)	-0.04 (-0.22,0.14)	-0.04 (-0.14,0.06)	-0.06 (-0.20,0.08)	-0.06 (-0.15,0.03)	-0.07 (-0.26,0.12)	-0.06 (-0.17,0.05)	-0.07 (-0.16,0.01)
0.02 (-0.06,0.11)	0.01 (-0.08,0.10)	**LR**	-0.01 (-0.24,0.21)	-0.03 (-0.21,0.14)	-0.03 (-0.13,0.06)	-0.05 (-0.19,0.09)	-0.05 (-0.14,0.04)	-0.06 (-0.25,0.12)	-0.05 (-0.17,0.06)	-0.07 (-0.15,0.02)
0.04 (-0.19,0.26)	0.02 (-0.20,0.25)	0.01 (-0.21,0.24)	**TIN**	-0.02 (-0.20,0.16)	-0.02 (-0.25,0.21)	-0.04 (-0.21,0.14)	-0.04 (-0.26,0.19)	-0.05 (-0.23,0.13)	-0.04 (-0.28,0.20)	-0.05 (-0.28,0.17)
0.06 (-0.12,0.23)	0.04 (-0.14,0.22)	0.03 (-0.14,0.21)	0.02 (-0.16,0.20)	**Inception**	0.00 (-0.18,0.18)	-0.02 (-0.13,0.09)	-0.02 (-0.19,0.16)	-0.03 (-0.16,0.09)	-0.02 (-0.22,0.17)	-0.03 (-0.21,0.14)
0.05 (-0.04,0.15)	0.04 (-0.06,0.14)	0.03 (-0.06,0.13)	0.02 (-0.21,0.25)	-0.00 (-0.18,0.18)	**NB**	-0.02 (-0.16,0.13)	-0.02 (-0.11,0.08)	-0.03 (-0.22,0.16)	-0.02 (-0.14,0.10)	-0.03 (-0.13,0.06)
0.07 (-0.06,0.21)	0.06 (-0.08,0.20)	0.05 (-0.09,0.19)	0.04 (-0.14,0.21)	0.02 (-0.09,0.13)	0.02 (-0.13,0.16)	**ResNet**	-0.00 (-0.14,0.14)	-0.01 (-0.14,0.11)	-0.00 (-0.16,0.16)	-0.02 (-0.15,0.12)
0.07 (-0.01,0.16)	0.06 (-0.03,0.15)	0.05 (-0.04,0.14)	0.04 (-0.19,0.26)	0.02 (-0.16,0.19)	0.02 (-0.08,0.11)	0.00 (-0.14,0.14)	**KNN**	-0.01 (-0.20,0.17)	-0.00 (-0.12,0.11)	-0.02 (-0.10,0.07)
0.09 (-0.10,0.27)	0.07 (-0.12,0.26)	0.06 (-0.12,0.25)	0.05 (-0.13,0.23)	0.03 (-0.09,0.16)	0.03 (-0.16,0.22)	0.01 (-0.11,0.14)	0.01 (-0.17,0.20)	**VGG**	0.01 (-0.19,0.21)	-0.00 (-0.19,0.18)
0.08 (-0.03,0.19)	0.06 (-0.05,0.17)	0.05 (-0.06,0.17)	0.04 (-0.20,0.28)	0.02 (-0.17,0.22)	0.02 (-0.10,0.14)	0.00 (-0.16,0.16)	0.00 (-0.11,0.12)	-0.01 (-0.21,0.19)	**DT**	-0.01 (-0.12,0.10)
**0.09 (0.01,0.17)**	0.07 (-0.01,0.16)	0.07 (-0.02,0.15)	0.05 (-0.17,0.28)	0.03 (-0.14,0.21)	0.03 (-0.06,0.13)	0.02 (-0.12,0.15)	0.02 (-0.07,0.10)	0.00 (-0.18,0.19)	0.01 (-0.10,0.12)	**SVM**

Data are differences in NPV (column-defining model minus row-defining model) with 95% confidence intervals. A positive value indicates that the column model has higher diagnostic performance than the row model. Bold font indicates statistical significance.

SVM, Support Vector Machine; RF, Random Forest; DT, Decision Tree; NB, Naive Bayes; LR, Logistic Regression; KNN, K-Nearest Neighbors; ResNet, Residual Network; Inception, Inception Network; VGG, Visual Geometry Group Network; TIN, Temporal Interlace Network; Boosting, Boosting-based algorithm models.

### Sensitivity and additional subgroup analyses

All patients included in this study underwent pathological examination to confirm axillary lymph node status. Specifically, 240 patients in Pengfei Sun et al. (21) and 597 patients in Zhang et al. (13) who underwent percutaneous needle biopsy, while all remaining patients (1,976 patients) underwent surgical pathology as the reference standard. Sensitivity analysis was performed by excluding 240 patients in Pengfei Sun et al. (21) and the 597 patients from Zhang et al. (13) who underwent percutaneous needle biopsy, while retaining the 305 surgical pathology patients from the same study of Zhang et al. (13). No full study was excluded. After this exclusion, the SUCRA rankings remained largely unchanged, with Boosting-based models still showing the highest SEN (SUCRA = 73.8) and NPV (SUCRA = 83.6). TIN maintaining the highest SPE (SUCRA = 78.9) and PPV (SUCRA = 81.5).

Sensitivity analysis treating each Boosting variant separately showed consistent rankings: XGBoost (SUCRA = 70.2), LightGBM (SUCRA = 68.5), and AdaBoost (SUCRA = 65.1) all maintained favorable performance, supporting the robustness of our grouping strategy.

The prevalence of ALNM across studies ranged from 33.3% to 55.6%. Meta-regression showed no significant association between ALNM prevalence and the diagnostic performance of the models (SEN: *P* = 0.21, SPE: *P* = 0.34), indicating that prevalence did not substantially affect the comparative rankings (see **Table 7**). Sensitivity analyses using alternative within-study correlation assumptions (ρ = 0.2, 0.6, 0.8) produced SUCRA rankings consistent with the primary analysis (ρ= 0.6). Boosting maintained the highest SEN SUCRA across all correlation strengths (ρ= 0.2: 72.5; ρ= 0.6: 71.9; ρ= 0.8: 71.2), and TIN maintained the highest SPE SUCRA (ρ= 0.2: 80.1; ρ= 0.6: 79.6; ρ= 0.8: 78.9), supporting the robustness of our findings to different correlation structures.

**Table 7:** Sensitivity and subgroup analysis.

Analysis	Subgroup	Studies (n)	Boosting SEN (SUCRA)	TIN SPE (SUCRA)
Main analysis	All studies	9	71.9	79.6
Exclude inconsistent reference	Studies with uniform reference (surgical pathology only)	8	72.3	79.6
By study design	Prospective studies	3	73.1	78.9
	Retrospective studies	6	71.2	80.1
By ALNM prevalence	Low prevalence (<40%)	3	72.5	78.2
	High prevalence (≥40%)	6	71.4	80.3

## Discussion

This study conducted a network analysis based on the predictive efficacy of multiple artificial intelligence models for ALNM in BC patients, aiming to explore the comparative diagnostic performance of multiple AI models and to identify potential signals of favorable predictive performance. The study found that all models demonstrated predictive efficacy for ALNM. Boosting-based algorithm models achieved the highest SEN (83.0%, 95% CI: 73.4-89.6%), and significantly higher sensitivity than SVM (difference: 0.17, 95% CI: 0.01-0.33). The predictive performance of the VGG and SVM models was relatively poor. The quality of the included studies was good, and no obvious publication bias was detected. Substantial heterogeneity was present (I² = 92%), and consistency tests revealed significant inconsistency (P < 0.05), likely due to differences in study populations, ultrasound protocols, or reference standards. These issues were partially addressed by using random-effects models and subgroup analyses, but the presence of significant inconsistency remains a major limitation.

Boosting-based algorithm models demonstrated good and balanced predictive performance across various diagnostic indicators. The pooled diagnostic accuracy analysis showed that Boosting achieved the highest pooled SEN (83.0%) and NPV (86.4%) among all models (see **Table 2**). Subgroup analysis (see **Table 3**) also confirmed that the Boosting-based groups had the highest pooled SEN (79.6%) and NPV (87.1%) compared with CNN-based and traditional ML groups. Due to the large number of Boosting-based algorithm models and their consistent theoretical basis, this study grouped them together for analysis. These models mainly include XGBoost, AdaBoost, LightGBM, and other related models.

Although XGBoost, AdaBoost, and LightGBM differ in specific optimization strategies, they share the core ensemble principle of iteratively training weak learners to correct prior errors. From a clinical prediction perspective, they also share similar input-output characteristics. Moreover, our sensitivity analysis treating each variant separately showed consistent SUCRA rankings (XGBoost: 70.2, LightGBM: 68.5, AdaBoost: 65.1), supporting the robustness of grouping these algorithms into a single ‘Boosting-based’ category for the primary analysis. Boosting class algorithms are a class of integrated learning methods, with the core idea of iteratively training multiple weak classifiers and weighting and combining them into a single strong classifier to significantly improve the overall predictive performance of the model (27, 28). Boosting algorithms deeply mine complex features by focusing on the errors and integrating the ability to strongly classify them to improve the ability to recognize tiny lesions in complex samples and reduce the number of under diagnosis. Boosting-based algorithms have demonstrated superior performance across multiple dimensions in human health (29–32). Numerous previous studies have demonstrated that Boosting-based algorithmic models, mainly XGBoost, have good predictive performance in terms of comprehensive BC influencing factors, diagnosis, recurrence, and prognosis (33–38). Some clinical experimental studies have affirmed the predictive value of Boosting-based models in ALNM, which is consistent with the results of this study. In addition, a study by Qishan Cen et al. found that the LightGBM algorithm demonstrated superior performance in mining BC ultrasound features in the peri-tumor region to construct a deep feature model for predicting the expression level of Ki-67 (AUC: 0.62, 95%CI: 0.55-0.70) (39). Another study found that the XGBoost model achieved an AUC of 0.94 in distinguishing luminal and non-luminal BC subtypes, respectively, and may also demonstrate better value in MRI imaging methods (40). Although the Boosting-based algorithmic model shows better predictive value in BC diagnosis and treatment, there is a contradiction between its basic mechanism of relying on high-quality features and data distribution and iteratively focusing on the wrong samples, and the clinical demands of less medical data, more noise, complex features, and high interpretability requirements. In the future, it is still necessary to optimize the model classification, improve the sample balancing strategy, and enhance the model interpretability in order to better fit the clinical scenarios.

In this study, the TIN model achieved a specificity of 90.0%, accuracy of 85.3%, and positive predictive value of 80.0% in predicting axillary lymph node metastasis in breast cancer patients. However, these results are based on a single study (n = 61) and require further validation. The TIN model has become one of the models for the analysis of video temporal information through the differentiable temporal offset operation. TIN was developed by 2020, and its core mechanism is to enable the interaction of information between neighboring frames by calculating the amount of bias of the target feature in the temporal dimension (17). This enables the TIN model to capture richer dynamic features and subtle changes, which in turn improves the specificity and accuracy of prediction. Although the lightweight structure of the TIN model replaces the explicit time dimension processing of traditional 3D convolution with non-explicit temporal modeling, it is simpler and more effective than the traditional 3D convolution in video classification models. However, its pooled diagnostic performance should be interpreted with caution. The high SPE (90.0%) and ACC (85.3%) in that single study may not be generalizable. Second, although TIN shows a good ACC (SUCRA = 77.4), its NPV (SUCRA = 50.9) is low, uneven results in predictive metrics. This phenomenon may lead to higher misclassification bias, resulting in underdiagnosis and inevitable under-treatment. Finally, in the clinical practice of US screening and prediction of BC patients, the model has high requirements in data acquisition and processing, and usually requires a larger sample size for model training. Therefore, its high diagnostic performance still needs further experimental validation.

The study by Li et al. (13) indicated that among CNN models, ResNet model showed the best performance (AUC = 0.86) for axillary lymph node metastasis prediction in BC patients (17). In contrast, in the results of this study, SVM and VGG exhibited relatively poor prediction performance. Factors such as the high dependence of traditional CNN models on artificial features, loss of subtle features, and insufficient processing of hard samples may be the reasons for their poor prediction ability. More flexible CNN architectures such as U-Net, ResNet, etc. may show relative advantages by preserving details and clinical features. In addition, the studies included in this study has less data comparing CNN-related models with other types of models. This seriously affects the interactivity and comparability of the comparisons between the models in the study. Large-model prospective clinical trial studies should be conducted to strengthen the direct link between CNN models and other types of artifacts and interactively explore the predictive value of different studies in ALNM.

The substantial heterogeneity and significant inconsistency observed in this analysis preclude current clinical recommendation. Nevertheless, our findings may offer valuable hypotheses to guide future research. If validated in prospective studies, Boosting-based algorithms might be explored as potential rule-out tools. These algorithms are characterized by their high sensitivity and high negative predictive value. They potentially help safely avoid unnecessary sentinel lymph node biopsies in low-risk patients, thereby reducing surgical complications and healthcare costs. Conversely, CNN-based models might assist in identifying high-risk patients who would benefit from axillary lymph node dissection. These models have high specificity and high positive predictive value. A complementary two-step workflow may be envisioned. This workflow would involve Boosting-based screening followed by CNN-based confirmation. It would enable rapid, non-invasive risk stratification using routine ultrasound images without additional procedures or costs. However, several challenges remain. Generalizability is a primary concern, as all included studies originated from Chinese hospitals. Model performance may vary across different populations, ultrasound devices, and imaging protocols. Interpretability also poses a barrier. Clinicians remain hesitant to trust “black-box” models without clear explanations. Prospective multi-center external validation studies are urgently needed before any clinical implementation can be considered.

Model fit assessment using AIC showed that the inconsistency model yielded a lower value than the consistency model (AIC: 142.3 vs. 156.8), indicating statistically significant inconsistency within the network. Despite this limitation, we exploratorily performed SUCRA ranking for reference. This approach provides a preliminary comparative signal that may generate hypotheses for future research. However, rankings derived under significant inconsistency may be unstable and subject to bias from network structure, and therefore should not be interpreted as definitive evidence of superiority of AI model.

Several factors may affect the transitivity and comparability assumptions underlying our network meta-analysis. The included studies varied in design, which may introduce bias. However, our subgroup analysis by study design showed consistent rankings across both subgroups, suggesting that design differences did not fundamentally alter the comparative performance of the models. Second, one study used mixed reference standards, while all other studies used surgical pathology alone. Sensitivity analysis excluding these study yielded similar rankings, indicating that this heterogeneity did not substantially affect the conclusions. ALNM prevalence varied across studies (33.3-55.6%), which could theoretically affect prevalence-dependent metrics (PPV, NPV). However, meta-regression showed no significant association between prevalence and diagnostic performance, and our primary conclusions are based on prevalence-independent metrics (SEN and SPE).

The study has the following limitations: first, the application of AI for clinical prediction in ultrasound was introduced relatively late, resulting in a small number of included studies and patients. Additional data are needed to strengthen the reliability of the conclusions. Second, substantial statistical heterogeneity (I² = 92%) and significant inconsistency between direct and indirect comparisons (P < 0.05) were present, indicating that the included studies may not be sufficiently comparable. Consequently, our SUCRA rankings and model comparisons should be viewed as hypothesis-generating rather than conclusive. Third, different AI models may capture different biological or imaging features from the same ultrasound data, and most pairwise comparisons lacked statistically significant differences, indicating substantial overlap in model performance. Additionally, our analysis approximated within-study correlations using assumed covariance structures, which may not fully capture the true correlation.

## Conclusion

In this network meta-analysis, Boosting-based algorithm models showed a possible signal of favorable and balanced predictive performance for ultrasound-detected ALNM, achieving the highest SEN (83.0%) and NPV (86.4%). However, due to substantial heterogeneity and inconsistency, these findings are hypothesis-generating. Future prospective studies are needed for validation.

## Data Availability

The original contributions presented in the study are included in the article/**Supplementary Material**. Further inquiries can be directed to the corresponding author.
