# Supplementary material for: The predictive value of multiple artificial intelligence models in axillary lymph node metastasis of breast cancer detected by ultrasound — a network meta-analysis
Source: Front Oncol. 2026 Jun 29;16:1725537. doi: 10.3389/fonc.2026.1725537 (PMC13357121; doi:10.3389/fonc.2026.1725537)
Supplement: Supplementary file 1 [file Table1.docx]

# **Supplementary Material 1: Full Reproducible Search Strategies for Each Database**

****Search period:**** July 1, 2015 – July 31, 2025
****Language restriction:**** None

## **Table S1. Search strategies for each database**

| **Database** | **Search Strategy** |
| --- | --- |
| ****PubMed**** | ("Artificial Intelligence"[MeSH] OR "Intelligence, Artificial"[Title/Abstract] OR "Computer Reasoning"[Title/Abstract] OR "Reasoning, Computer"[Title/Abstract] OR "AI (Artificial Intelligence)"[Title/Abstract] OR "Machine Intelligence"[Title/Abstract] OR "Intelligence, Machine"[Title/Abstract] OR "Computational Intelligence"[Title/Abstract] OR "Intelligence, Computational"[Title/Abstract] OR "Computer Vision Systems"[Title/Abstract] OR "Computer Vision System"[Title/Abstract] OR "System, Computer Vision"[Title/Abstract] OR "Systems, Computer Vision"[Title/Abstract] OR "Vision System, Computer"[Title/Abstract] OR "Vision Systems, Computer"[Title/Abstract] OR "Knowledge Acquisition (Computer)"[Title/Abstract] OR "Acquisition, Knowledge (Computer)"[Title/Abstract] OR "Knowledge Representation (Computer)"[Title/Abstract] OR "Knowledge Representations (Computer)"[Title/Abstract] OR "Representation, Knowledge (Computer)"[Title/Abstract]) AND ("Axillary lymph nodes"[MeSH] OR "axillary lymph node"[Title/Abstract] OR "axillary lymph nodes"[Title/Abstract] OR "axillary metastasis"[Title/Abstract] OR "axillary lymph node metastasis"[Title/Abstract]) AND ("Ultrasonography"[MeSH] OR "Diagnostic Ultrasound"[Title/Abstract] OR "Diagnostic Ultrasounds"[Title/Abstract] OR "Ultrasound, Diagnostic"[Title/Abstract] OR "Ultrasounds, Diagnostic"[Title/Abstract] OR "Echography"[Title/Abstract] OR "Echotomography"[Title/Abstract] OR "Sonography, Medical"[Title/Abstract] OR "Medical Sonography"[Title/Abstract] OR "Ultrasonic Imaging"[Title/Abstract] OR "Imaging, Ultrasonic"[Title/Abstract] OR "Ultrasonographic Imaging"[Title/Abstract] OR "Imaging, Ultrasonographic"[Title/Abstract] OR "Ultrasound Imaging"[Title/Abstract] OR "Imaging, Ultrasound"[Title/Abstract] OR "Computer Echotomography"[Title/Abstract] OR "Ultrasonic Tomography"[Title/Abstract] OR "Ultrasonic Diagnosis"[Title/Abstract]) AND ("2015/07/01"[Date - Publication] : "2025/07/31"[Date - Publication]) |
| ****Embase**** | ('artificial intelligence'/exp OR 'machine intelligence':ab,ti OR 'artificial intelligence':ab,ti OR 'computer reasoning':ab,ti OR 'computational intelligence':ab,ti OR 'computer vision systems':ab,ti OR 'knowledge acquisition':ab,ti) AND ('axillary lymph node'/exp OR axillary AND ('lymph'/exp OR lymph) AND nodes OR 'axillary lymph node metastasis':ab,ti) AND ('echography'/exp OR 'diagnostic ultrasound':ab,ti OR 'diagnostic ultrasonic examination':ab,ti OR 'diagnostic ultrasonic imaging':ab,ti OR 'ultrasonography':ab,ti OR 'sonography':ab,ti OR 'ultrasound diagnosis':ab,ti OR 'ultrasonic diagnosis':ab,ti) AND [2015-2025]/py |
| ****Web of Science**** | TS=("artificial intelligence" OR "intelligence, artificial" OR "computer reasoning" OR "reasoning, computer" OR "machine intelligence" OR "computational intelligence" OR "computer vision systems" OR "knowledge acquisition") AND TS=("axillary lymph node*" OR "axillary metastasis" OR "axillary lymph node metastasis") AND TS=("ultrasonography" OR "diagnostic ultrasound" OR "echography" OR "sonography" OR "ultrasonic imaging" OR "ultrasound imaging") AND DT=(Article OR Review OR Clinical Trial) AND PY=(2015-2025) |
| ****Cochrane Library**** | ("artificial intelligence" OR "machine intelligence" OR "computer reasoning" OR "computational intelligence" AND "ultrasonography" OR "diagnostic ultrasound") AND ("axillary lymph node" OR "axillary lymph node metastasis" OR "axillary metastasis") AND ("breast cancer") |
| ****CNKI (China National Knowledge Infrastructure)**** | (主题='人工智能' OR '机器学习' OR '深度学习' OR '计算机视觉' OR '超声' OR '超声成像') AND (主题='腋窝淋巴结' OR '腋淋巴结转移' OR '腋窝淋巴结转移') AND (发表时间 between 2015-07-01 and 2025-07-31) |
| ****Wanfang Data**** | 主题:(人工智能 or 机器学习 or 深度学习 or 超声 or 超声成像) and 主题:(腋窝淋巴结 or 腋淋巴结转移) Date:2015-2025 |

Purpose of this document:

This supplementary material provides the complete, reproducible search strategies for all six databases used in our network meta-analysis. These strategies were originally executed on July 25, 2025, and cover the period from July 1, 2015, to July 31, 2025. No post-hoc modifications were made to the search strings. The strategies are reported in accordance with the PRISMA-NMA statement to ensure transparency and replicability.
